# Long-term outcomes of oral rehabilitation with dental implants in HIV-positive patients: A retrospective case series

**DOI:** 10.4317/medoral.21028

**Published:** 2016-03-06

**Authors:** Cosme Gay-Escoda, Débora Pérez-Álvarez, Octavi Camps-Font, Rui Figueiredo

**Affiliations:** 1 MD, DDS, MS, PhD. Professor and Head of Oral and Maxillofacial Surgery, Faculty of Dentistry, University of Barcelona. Coordinating researcher at the IDIBELL institute. Head of the Oral and Maxillofacial Surgery Department, Teknon Medical Center, Barcelona, Spain; 2 DDS, MS. Master in Oral Surgery and Implantology. Faculty of Dentistry, University of Barcelona, Spain; 3 DDS. Specialty registrar, Master in Oral Surgery and Implantology degree course, Faculty of Dentistry, University of Barcelona, Spain; 4 DDS, MS, PhD. Associate lecturer in Oral Surgery and lecturer on the Master of Oral Surgery and Implantology degree course, Faculty of Dentistry, University of Barcelona, Spain. Researcher at the IDIBELL Institute

## Abstract

**Background:**

The existing information on oral rehabilitations with dental implants in VIH-positive patients is scarce and of poor quality. Moreover, no long-term follow-up studies are available. Hence, the aims of this study were to describe the long-term survival and success rates of dental implants in a group of HIV-positive patients and to identify the most common postoperative complications, including peri-implant diseases.

**Material and Methods:**

A retrospective case series of HIV-positive subjects treated with dental implants at the School of Dentistry of the University of Barcelona (Spain) was studied. Several clinical parameters were registered, including CD4 cell count, viral load and surgical complications. Additionally, the patients were assessed for implant survival and success rates and for the prevalence of peri-implant diseases. A descriptive statistical analysis of the data was performed.

**Results:**

Nine participants (57 implants) were included. The patients’ median age was 42 years (IQR=13.5 years). The implant survival and success rates were 98.3% and 68.4%, respectively, with a mean follow-up of 77.5 months (SD=16.1 months). The patient-based prevalence of peri-implant mucositis and peri-implantitis were 22.2% and 44.4% respectively at the last appointment. Patients that attended regular periodontal maintenance visits had significantly less mean bone loss than non-compliant patients (1.3 mm and 3.9 mm respectively).

**Conclusions:**

Oral rehabilitation with dental implants in HIV-positive patients seems to provide satisfactory results. In order to reduce the considerably high prevalence of peri-implant diseases, strict maintenance programmes must be implemented.

**Key words:**HIV infection, dental implants, oral implantology, complications, peri-implantitis, peri-implant diseases.

## Introduction

Acquired immune deficiency syndrome (AIDS) is a condition caused by the human immunodeficiency virus (HIV). In 2012 it affected nearly 30 million people worldwide (1). Patients suffering from AIDS experience an immune depression, caused by HIV infection, which reduces the host’s resistance to pathogens. An HIV infected patient is seropositive but will only develop AIDS symptoms when the CD4 T-helper lymphocyte count (CD4) is less than 200 cells/mm (2). Oral manifestations of AIDS include oral candidiasis, hairy leukoplakia, Kaposi’s sarcoma, HIV-associated gingivitis and periodontitis, atypical ulcerations and herpetic infections (3).

The introduction of highly active antiretroviral therapy (HAART) in 1996 has reduced HIV-related morbidity and mortality significantly in areas with access to antiretrovirals (2), converting this disease into a chronic condition. HAART includes a combination of antiretroviral medications, such as nucleoside reverse transcriptase inhibitors (NRTIs), non-nucleoside reverse transcriptase inhibitors (NNRTIs), protease inhibitors (PIs), entry inhibitors and integrase inhibitors (4). However, these drugs also have some adverse effects, including diarrhea, anaemia, dyslipidaemia, pancreatitis, hepatotoxicity, hyposalivation and bone metabolism disorders like osteopenia, osteonecrosis and osteoporosis (4,5). Nevertheless, these effective treatments can keep patients asymptomatic for a long period of time. Accordingly, demands for functional and aesthetic dental treatments have increased in recent years. In this context, oral rehabilitation with dental implants can be a good alternative to traditional removable prostheses. Although no long-term longitudinal studies have been published on this subject, several papers have suggested that immunologically stable patients might be treated successfully with implant-prosthetic rehabilitation (6-13). On the other hand, some studies have shown that when the CD4 count is <200 cells/3, the risk of postoperative complications is high (14).

The aims of this study were to describe the long-term survival and success rates of dental implants in a group of HIV-positive patients and to quantify their most common postoperative complications, including peri-implant diseases.

## Material and Methods

A retrospective case series of HIV-positive patients consecutively treated with dental implants between July 2004 and May 2008 in the Oral Surgery and Implantology Unit of the School of Dentistry of the University of Barcelona was studied.

The study design followed the STROBE guidelines for cohort studies (15). The protocol complied with the Helsinki declaration guidelines and was approved by the Ethical Committee for Clinical Research (CEIC) of the Dental Hospital of the University of Barcelona.

The patients were given full information about the surgical procedures and treatment alternatives and duly signed informed consent forms. Preoperative analysis included complete medical histories and clinical and radiographic examinations (with panoramic radiographs and/or computed tomographic scans).

At the time of implant surgery, all patients presented an American Society of Anesthetists (ASA) health status score of no higher than 3. Patients with active periodontal disease had been treated previously according to the American Academy of Periodontology guidelines (16). Patients with incomplete clinical records were excluded from the analysis.

All the participants were recalled for a follow-up visit in 2014, which included periodontal and peri-implant clinical and radiological examinations, in order to assess their implant survival and success rates, measured according to the criteria of Albrektsson *et al.* (17).

- Surgical Procedure

Preoperative consultation with the infectious diseases specialist was required in all cases. Recent blood tests, dating from less than 2 months previously – including basic haemostasis profile, blood biochemistry, haematic biometry, lymphocyte sub-type counts (CD4, CD8, CD4:CD8 ratio, NK) and viral load – were mandatory before the surgical procedure.

Final-year postgraduates on the 3-year Master in Oral Surgery and Implantology degree course placed the implants under local anesthesia, generally a 4% solution of articaine with 1:100.000 epinephrine, with or without simultaneous (multimodal) intravenous conscious sedation with midazolam 2 mg; propofol 0.3-0.5 mg/kg/h and remifentanil 0.025-0.05 mcg/kg/min. One hour before the surgical procedure, the patients received 2g of amoxicillin. A midcrestal incision was made and full-thickness flaps were raised to expose the alveolar ridge. The implant sites were prepared using drills of increasing diameter under constant irrigation with sterile saline solution, following the manufacturers’ recommendations. Functional and aesthetic requirements were taken into account in determining the mesiodistal and buccolingual inclination of the implants and the need for guided bone regeneration (GBR) procedures. The flaps were usually repositioned with 4-0 Supramid sutures (Supramid®; Aragó, Barcelona, Spain). The suture was removed 7 to 15 days after surgery.

After the operation, an antibiotic (usually amoxicillin 750 mg every 8 hours for 7 days), a nonsteroidal anti-inflammatory drug (usually sodium diclofenac 50 mg every 8 hours for 4-5 days), an analgesic (usually paracetamol 1 g every 8 hours for 3-4 days), and a mouthrinse (0.12% chlorhexidine digluconate 15 ml every 12 hours for 15 days) were prescribed.

The postoperative instructions and prescribed drugs were explained verbally and on a sheet of paper that was given to the patient. The patients’ compliance was not specifically assessed.

The patients were recalled for a periodontal and peri-implant maintenance visit at least once a year. Every follow-up appointment consisted of oral hygiene instructions, prosthesis maintenance and a complete periodontal examination which checked the probing pocket depth (PPD), the modified plaque and gingival index according to Mombelli *et al.* (18) (mPI and mGI), bleeding on probing (BoP) and suppuration.

During the latest follow-up visit, periapical digital radiographs using long-cone parallel technique X-rays were obtained in order to measure the bone levels (BL). A single researcher (OCF) assessed the mesial and distal aspects of each implant and recorded the highest value. All the radiographs were examined twice, 1 week apart, to verify intra-examiner reproducibility. The intraclass correlation coefficient (ICC) was 0.908 (95% CI: 0.867-0.941; *p*<0.001), indicating nearly perfect agreement.

Each implant was assessed for the presence of peri-implant diseases, applying the following diagnostic criteria suggested by Koldsland *et al.* (19):

- Health: BL < 2 mm with mGI = 0

- Inflammation: any BL with mGI ≥ 1

* Peri-implant mucositis: BL < 2 mm with mGI ≥ 1

* Peri-implantitis: BL ≥ 2 mm with mGI ≥ 1 or suppuration.

For a patient to be considered healthy, all his or her implants had to be classified as healthy. If any of the implants was classified into the mucositis or peri-implantitis groups, the patient was considered not healthy. The patients were classified into the group of their worst implant. Specific treatment for peri-implant diseases was given if needed.

Compliance with periodontal maintenance was defined as attendance at more than two-thirds of the scheduled visits.

- Data sampling

All the clinical records were examined by the same researcher (OCF), who collected the following data: age; gender; smoking habit (non-smoker, 1 to 10 cigarettes/day, or more than 10 cigarettes/day); drug habits; year of HIV infection diagnosis; HIV transmission route (sexual, parenteral or transfusion); CD4 lymphocyte count; viral load; antiretroviral therapy; periodontal disease (healthy or periodontally compromised); quality and quantity of available alveolar bone using the most widely accepted classification according to Ribeiro-Rotta *et al.* (20); implant manufacturer; location (maxilla or mandible); position (anterior or posterior); primary stability (considered when insertion torque was over 15 N·cm2); intra and/or postoperative complications; time from implant placement to prosthetic loading (immediate: within the first week; early: between 1 week and 3 months in the mandible and 1 week and 4 months in the maxilla; conventional, at least 3 months in the mandible and 4 months in the maxilla); type of prosthetic restoration; date of the most recent follow-up; and peri-implant parameters (BoP, PPD, mGI, BL and suppuration).

- Statistical analysis

The statistical analysis was carried out with the Statistical Package for the Social Sciences (SPSS version 22.0; IBM Corp.; Armonk, NY, USA). The normality of the scale variables (patient age, CD4 cell count, PPD, time elapsed from implant placement to prosthetic loading and follow-up period) was explored using the Shapiro-Wilks test. Where normality was rejected, the interquartile range (IQR) and median were calculated. Where distribution was compatible with normality, the mean and standard deviation (SD) were used. The level of significance was set at *p*<0.05. Ninety-five percent confidence intervals (95% CI) were calculated for all prevalences.

## Results

Fifty-seven dental implants were placed in 9 patients who met the inclusion criteria.

The median age of the patients was 42 years (IQR=13.5 years). There were 5 males (55.6%) and 4 females (44.4%). Six patients (66.7%) were smokers, 2 (22.2%) were regular cannabis smokers and 4 (44.4%) had a history of intravenous drug dependence. The median CD4 cell count was 436.0 cells/mm3 (IQR=606.5 cells/mm3) and viral load values were undetectable (<50 copies/mL) in 8 patients (88.9%).  Table 1 shows the main features of the sample.

**Table 1:**
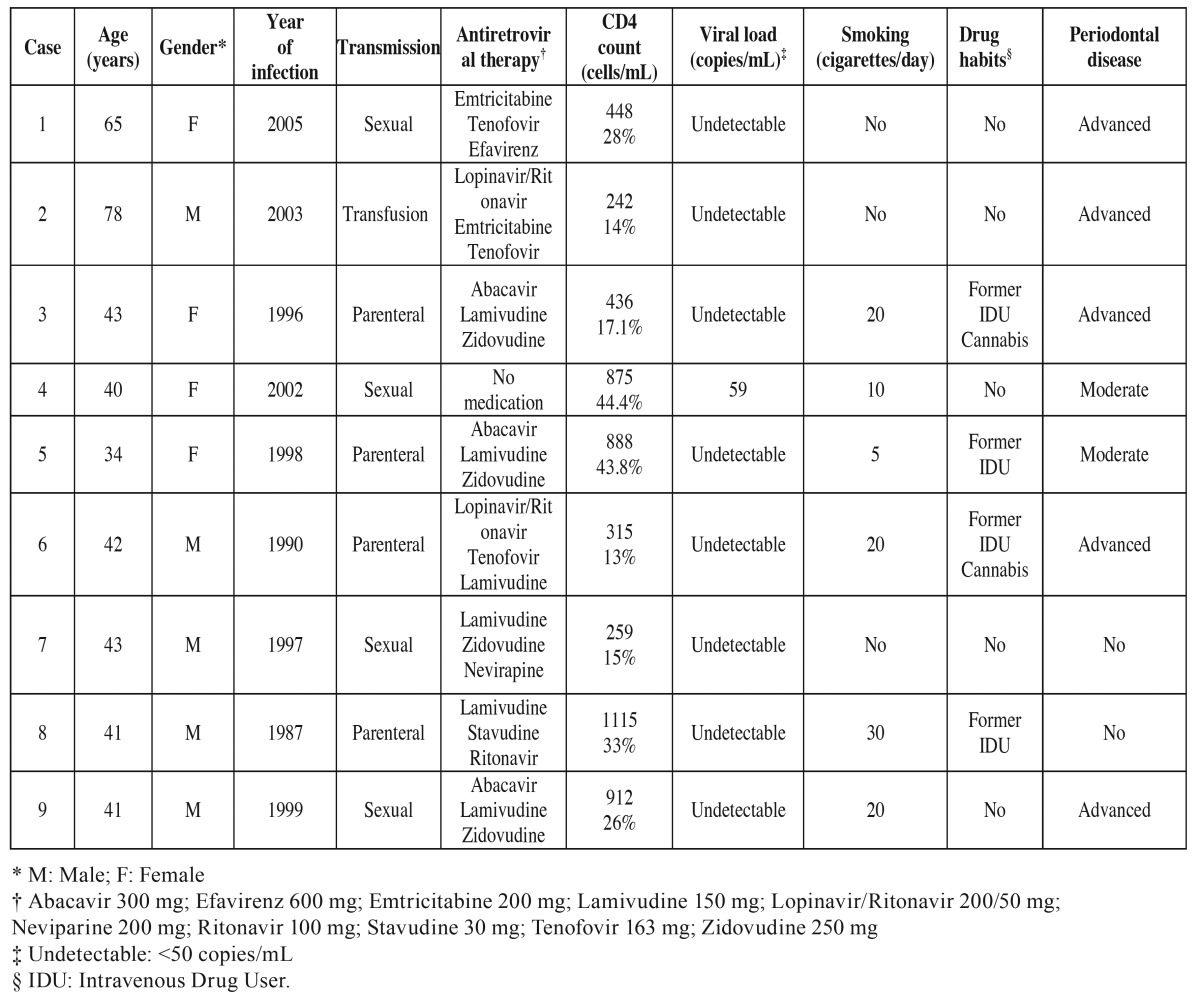
Main demographic characteristics of the patients.

All the implants were inserted at least 4 months after tooth extraction. GBR procedures were required in cases 1 (simultaneously) and 3 (prior to implant placement). Neither intra- nor early post-operative complications were reported. All the implants were conventionally loaded with the exception of case 4 (immediate loading).

The implant survival and success rates were 98.3% (one implant failed in case 3 due to peri-implantitis) and 68.4% respectively, with a mean follow-up period of 77.5 months (SD=16.1 months).  Table 2 describes some of the HIV-patients’ clinical features and treatment outcomes.

**Table 2:**
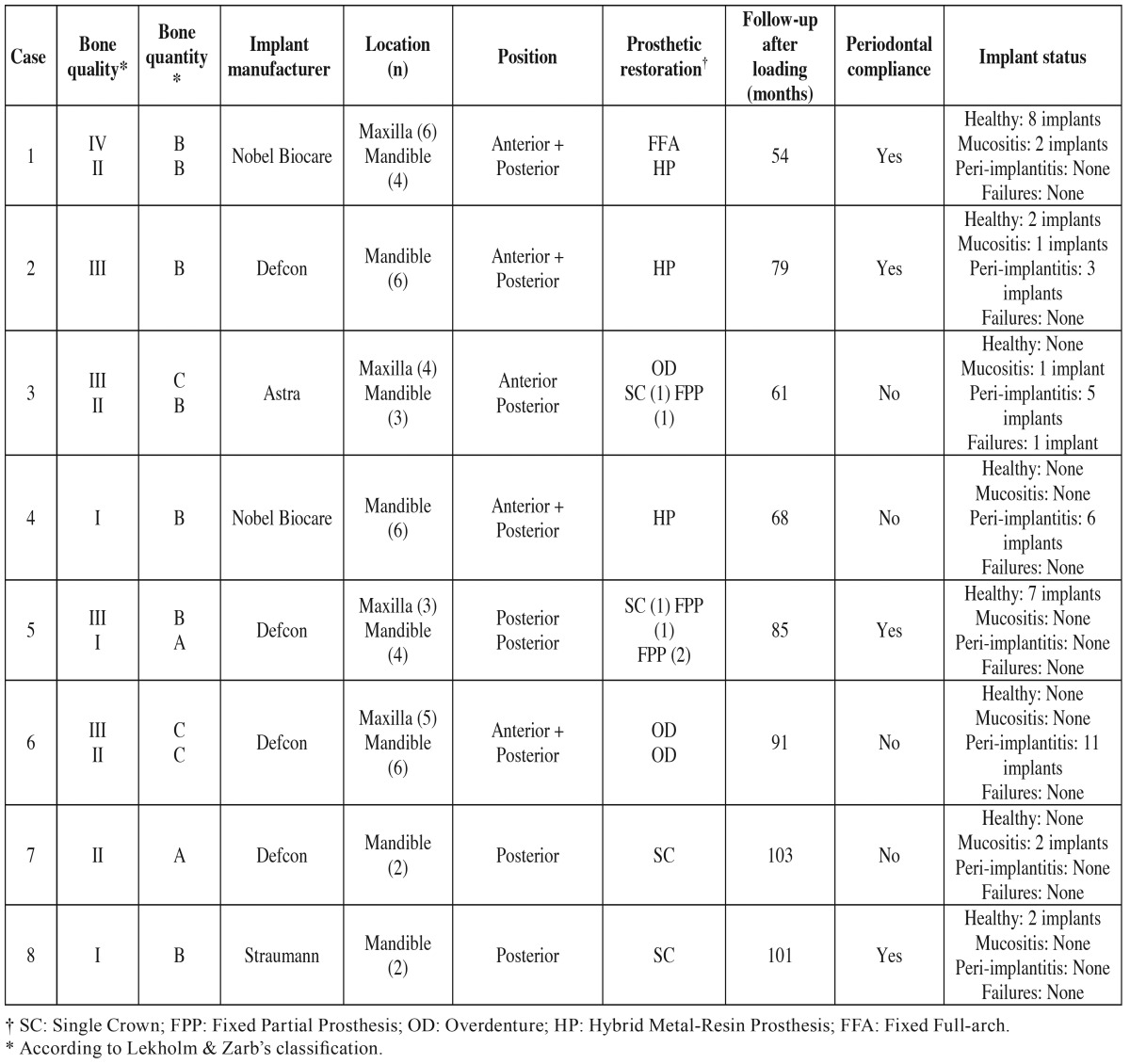
Clinical features and treatment outcomes.

Six implants (2 patients) presented mucositis and 26 implants (4 patients) were diagnosed with peri-implantitis. The patient and implant-based prevalences of these complications were 22.2% (95%CI: 6.3% to 54.7%) and 10.5% (95%CI: 4.9% to 21.1%) for mucositis and 44.4% (95%CI: 18.9% to 73.3%) and 45.6% (95%CI: 33.4% to 58.4%) for peri-implantitis.  Table 3 summarizes the peri-implant examination results at the most recent follow-up appointment. Only 1 of the 5 patients that complied with the periodontal/peri-implant maintenance visits had peri-implantitis, whereas all the non-compliant patients were classified as not healthy (3 had peri-implantitis and 1 had mucositis) ( Table 1- Table 3).

**Table 3:**
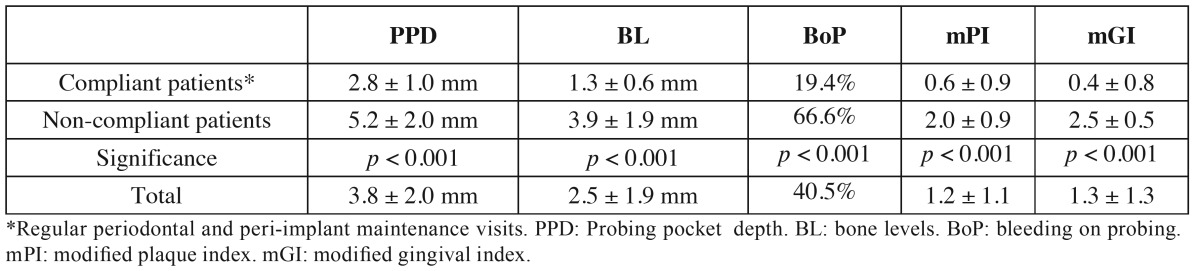
Peri-implant parameters at the most recent follow-up appointment.

## Discussion

The present study, conducted after 5 to 9 years of follow-up, indicates that oral rehabilitation with dental implants is a valid treatment option for HIV-positive patients.

A commonly accepted surgical principle is that immunocompromised patients have an increased risk of postoperative complications due to their inability to generate a controlled, appropriate and sustained immune response (21). However, even though literature on this subject is scarce, all the studies published reveal that implant placement in immunologically stable HIV-positive patients does not increase the risk of developing postoperative complications such as infection or wound-healing impairment (6-12). Although Patton *et al.* (14) found significantly higher rates of postoperative complications following tooth extractions when pronounced immunosuppression (CD4 cell count <200 cells/mm3) and severe neutropenia (neutrophilic leukocytes <500 cells/3) were recorded, most authors have found no association between HIV infection and the occurrence of postoperative problems with other minor oral surgery procedures (21-23). In the present case series, no early postoperative complications were reported. However, the degree of immunosuppression seems to be an important variable and further research is needed to corroborate these findings.

The major limitations of the present study are its retrospective nature and the fact that several non-calibrated dentists conducted the periodontal/peri-implant maintenance visits. In order to limit the effect of these biases, a single researcher made the final assessment of all the cases, enabling objective data to be gathered on the main outcome variables (success and survival rates and peri-implant disease parameters). The low number of patients included in this study can also be considered a limitation. However, taking into account the lack of large-sample studies published in the literature on this subject and the long-term data this study provides, we think that the information reported in this paper will prove extremely interesting and useful to clinicians.

Baron *et al.* (6) suggested that a temporary reduction in CD4 cell counts after implant placement may happen due to an inflammatory process at the surgery site. This was not the case here, or in most of the studies cited (7-12), since no alterations in CD4 cell count or in other analytical parameters were observed during the follow-up period.

Although several authors claim that conventional protocols should be maintained in cases of immunological stability (6-12), most clinicians systematically prescribe both preoperative and postoperative antibiotics when invasive dental procedures are performed in HIV-positive patients (24). Hence, most administer systemic antibiotics (usually amoxicillin) during the first 5 to 7 days after implant placement (6-11). Further research on this topic is needed to confirm which regime is the most suitable for immunocompromised patients.

Dental implants are considered a safe and predictable treatment method with high rates of both survival and success after 5 and 10 years (25,26). Although the present findings confirm high long-term implant survival rates (98%), only 68% fulfilled the Albrektsson *et al.* (17) success criteria (27-29). This might be associated with the features of the sample, since there was a high rate of patients who smoked and also had a previous history of periodontitis, both of which are strong risk indicators for peri-implant pathology (30). Furthermore, several patients failed to attend regular periodontal maintenance visits. Indeed, an examination of this variable ( Table 2) shows that the 4 non-compliant patients were diagnosed with peri-implantitis (in 3 cases) or with mucositis (1 patient), whereas the remaining 5 patients, who attended the follow-up visits, had considerably less peri-implant disease (3 were healthy, 1 had mucositis and 1 was diagnosed with peri-implantitis). Moreover, significantly better results for all the peri-implant parameters were reported in the compliant patients ( Table 3) (31). Obviously, HIV infection might also play an important role in these complications. In fact, several periodontal lesions have been described in HIV positive patients (3), including linear gingival erythema and necrotizing periodontal diseases, as well as possible exacerbation of pre-existing periodontal diseases. All these factors highlight the need to implement strict maintenance protocols to prevent the onset and progression of peri-implant diseases in these patients.

In conclusion, oral rehabilitation with dental implants in HIV-positive patients with CD4+ cell counts of >200/µL and undetectable viral loads seems to provide satisfactory results. In order to reduce the very high prevalence of peri-implant diseases in HIV positive patients, strict periodontal maintenance programmes must be implemented.
